# Supplementary material for: Online searches of children’s oseltamivir in public primary and specialized care: Detecting influenza outbreaks in Finland using dedicated databases for health care professionals
Source: PLoS One. 2022 Aug 5;17(8):e0272040. doi: 10.1371/journal.pone.0272040 (PMC9355218; doi:10.1371/journal.pone.0272040)
Supplement: Attachment — Submitted filename: Review_PONE-D-21-11786.docx [file pone.0272040.s002.docx]

Dear editor,

Thank you for providing me with this opportunity to review the mmanuscript titled “Healthcare professionals’ online searches of children’s oseltamivir in primary and specialized care” submitted to Plos One journal.

In their interesting work, authors have focused on trying to establish a relationship between the number of searches in the primary and specialized care for oseltamivir and the recorded cases of flu cases in children. This study is valuable because the findings can potentially contribute to the effective prediction of flu pandemics using the frequency of searches for a specific term/phrase.

I believe that this manuscript can be considered for further review with additional work, and I do not suggest publication in its current form. I have made detailed comments related to each section of the manuscript below. This manuscript presents a lack of rigorous statistical analysis and an underdeveloped methodology, which need special attention.

# Title:

Suggestions: Change the title to something more generalizable that shows the usefulness of online search results for flu outbreak detection/prediction

# Keywords

Correct “speralized care” to “Specialized care”

# Abstract

Please consider these comments for the abstract and apply those to the body of the manuscript wherever applicable.

Well-constructed and organized, overall. Important notes:

Lines 24-30: The introductory sentences in the “Objective” section do not align correctly with the research objective. More specifically, the last sentence needs to be changed to reflect the objective of this research (closer to the “Rationale”).

Lines 34-38: Include the source of information/ data for both online searches and the recorded number of cases.

Lines 39-44: Results need some rigorous statistical analysis. At least, the correlation between the number of searches and the number of recorded cases should be considered. Moreover, if possible additional variables for regression analysis are suggested. Probably a formula can be developed to predict the number of cases using the frequency of searches.

Lines 45-46: The conclusion is stated in a form that suggests a binary outcome/incident as yes/no. It should be rephrased as a directional association. More searches= more reported cases (A positive correlation between the frequency of searches and the # of reported cases)

# Introduction

Overall the content of the introduction section is satisfactory. However, the organization and flow are relatively heavy to digest. Please consider using different paragraphs for each of the following content in the following order:

1- Influenza (include # of annual cases in Finland (or Prevalence rate), deaths, plus the burden of disease in terms of cost to the healthcare system.

2- Different options for Online searches, pros cons.

3- Details about the setting (Finish healthcare system) relevant to the topic.

4- The rationale for the need for this research and its expected value added to literature/practice.

# Material and Methods

This section is poorly developed and need much work, including:

1- Suggest adding/ or moving some content from the introduction and adding a “Setting” sub-heading to discuss the study setting.
2- The data sources for online searches and recorded cases should be included here.
3- The rationale and logic behind choosing this medication (oseltamivir) and why this one(and not other medications/ keywords?) should be clarified.
4- Important: The analysis is insufficient. At least, the correlation between the number of searches and the number of recorded cases should be considered. Moreover, if possible additional variables for regression analysis are suggested. Probably a formula can be developed to predict the number of cases using the frequency of searches.

Line 102: change “laboratory positive findings” to “positive laboratory findings”

# Results

The results section needs additional work.

Important: As a result of substantial changes suggested to the “Methods” section, the “Results” section also needs more development. Begin by including the descriptive statistics about the # of searches. Then, expand with correlation/regression findings. Some charts can be useful here

Line 114 and elsewhere: The season 2012/13 needs to be written transparently. For example, the phrase “In specialized care 2012/13 and 2013/14,” is confusing. Please use year and season to make these statements easy to understand.

Line 115: Please rewrite this phrase “a wider scale of months before and after these seasons”. It’s a bit unclear.

# Discussion

The discussion section talks about the potential reasons behind why searches are more/less in primary/specialized settings and probably why the reported cases are different in these settings. However, there is no discussion about the uses of these search result for prediction and potential confounders that can impact the accuracy/ issues of using these search results to predict the number of cases. Moreover, other studies/ applications similar to this research should be included and discussed here.

Lines 140-141: This statement does not reflect the relative size of the primary and specialized care. The total number of practitioners in different levels (primary/specialized care) should be included to make a legitimate comparison. More can be discussed here by knowing the size of each care level. Needs to be corrected “However, our study found that professionals in primary care use PD as much as their colleagues in specialized care”

# Conclusions

Change based on the results from additional statistical analysis leaned toward the prediction power of the search for oseltamivir in actual flu cases.

The suggestion for future research in other diseases is proper.
