# Supplementary material for: Online searches of children’s oseltamivir in public primary and specialized care: Detecting influenza outbreaks in Finland using dedicated databases for health care professionals
Source: PLoS One. 2022 Aug 5;17(8):e0272040. doi: 10.1371/journal.pone.0272040 (PMC9355218; doi:10.1371/journal.pone.0272040)
Supplement: Attachment — Submitted filename: Response to Reviewers.docx [file pone.0272040.s004.docx]

REBUTTAL LETTER

**Academic editor:** We have the response from the reviewers assigned for your revised manuscript. Overall, reviewers indicated that the revisions made by the authors addressed most of the questions raised by the first two reviewers. Moreover, I reviewed your revised manuscript, and I believe that with minor revisions, it can be considered for publication
Please consider the points made by the new reviewer (#4) and submit a revised version of your manuscript. Please also double-check your manuscript for the English language and proofread it thoroughly.

**Response:** Thank you for your comment. We have answered the points made by the reviewer #4 (please see below). The text has been language-checked, edited, and proofread in terms of proper English language.

**Academic editor:** Please review your reference list to ensure that it is complete and correct. If you have cited papers that have been retracted, please include the rationale for doing so in the manuscript text, or remove these references and replace them with relevant current references. Any changes to the reference list should be mentioned in the rebuttal letter that accompanies your revised manuscript. If you need to cite a retracted article, indicate the article’s retracted status in the References list and also include a citation and full reference for the retraction notice.

**Response:** We have checked the Reference list to ensure that it is complete and correct. Due to additional information embedded in Discussion, three references [33,34,35] have been added on the Reference list.

Reviewers' comments

Reviewer's Responses to Questions

**Comments to the Author**

1. If the authors have adequately addressed your comments raised in a previous round of review and you feel that this manuscript is now acceptable for publication, you may indicate that here to bypass the “Comments to the Author” section, enter your conflict of interest statement in the “Confidential to Editor” section, and submit your "Accept" recommendation.

Reviewer #3: (No Response)

Reviewer #4: All comments have been addressed

**Response 1:** Thank you for your comment.

2. Is the manuscript technically sound, and do the data support the conclusions?

The manuscript must describe a technically sound piece of scientific research with data that supports the conclusions. Experiments must have been conducted rigorously, with appropriate controls, replication, and sample sizes. The conclusions must be drawn appropriately based on the data presented.

Reviewer #3: (No Response)

Reviewer #4: Partly

**Response 2:** We have now added more information on conclusions supported by the data in the Discussion section. Please see below (Response 6).

3. Has the statistical analysis been performed appropriately and rigorously?

Reviewer #3: (No Response)

Reviewer #4: Yes

**Response 3:** Thank you for your comment.

4. Have the authors made all data underlying the findings in their manuscript fully available?

The [PLOS Data policy](http://www.plosone.org/static/policies.action#sharing) requires authors to make all data underlying the findings described in their manuscript fully available without restriction, with rare exception (please refer to the Data Availability Statement in the manuscript PDF file). The data should be provided as part of the manuscript or its supporting information, or deposited to a public repository. For example, in addition to summary statistics, the data points behind means, medians and variance measures should be available. If there are restrictions on publicly sharing data—e.g. participant privacy or use of data from a third party—those must be specified.

Reviewer #3: (No Response)

Reviewer #4: Yes

**Response 4:** Thank you for your comment.

5. Is the manuscript presented in an intelligible fashion and written in standard English?

PLOS ONE does not copyedit accepted manuscripts, so the language in submitted articles must be clear, correct, and unambiguous. Any typographical or grammatical errors should be corrected at revision, so please note any specific errors here.

Reviewer #3: (No Response)

Reviewer #4: No

**Response 5:** Thank you for pointing out your concern. The text has been language-checked, edited, and proofread in terms of proper English language.

6. Review Comments to the Author

Please use the space provided to explain your answers to the questions above. You may also include additional comments for the author, including concerns about dual publication, research ethics, or publication ethics. (Please upload your review as an attachment if it exceeds 20,000 characters)

Reviewer #3: (No Response)

Reviewer #4: This article provides the use of prescription antiviral dose look-up as a novel method for surveillance of influenza in children, and is an important and potentially helpful use of understanding influenza in our young populations.

**There could be more caveats provided around the possible confounders in these methodology: what other conclusions could be drawn from these data? are there any biases which arise from these data which have not been shared? and if so , what direction do they draw the results in? For example the difference in 1.7 in lab diagnostics between settings, but the dose checking was the same- what does this mean? what other explanations are there for this? Could anyone be looking up the does of osteltamivir for other reasons (ie teaching/learning?) Could people be prescribing without looking up the does once they know it?

The abstract doesn't read properly, and could do with checking for basic English.

However, this is a novel and important finding, and useful for surveillance of influenza in children, and is useful to share as helpful methods for public health practice.**

**General response 6:** Thank you for your comments. We have now added more information regarding your suggestions and answered them (and added in the manuscript) in detail below. Also, we have restructured the Discussion section due to the additional information embedded.

**Comment:** What other conclusions could be drawn from these data? Are there any biases which arise from these data which have not been shared? And if so, what direction do they draw the results in?

**Response:** Thank you for your important comment. We have gone through the discussion among co-authors, and we believe that the confounders and biases are now described in the Discussion section. We have now added the following sentences about these possible confounders in our study:

“In addition, HCPs other than physicians (e.g., nurses, pharmacists) may have searched for information on oseltamivir. However, we assume that the majority of oseltamivir searches occur in practice in primary and specialized care by clinical physicians.”

“There may be a wide variation of reporting diagnoses in distinct primary and specialized care units nationwide.”

“Third, while more likely to occur in the general population, the media coverage on the start of influenza season or other influenza-related news during the season may affect information seeking behavior among HCPs, thus showing the increase in oseltamivir searches. However, we did not measure media coverage and its potential impact on searches.”

“It is important to note that some parents may request a doctor to prescribe oseltamivir regardless of a test result. Due to these occasional cases, some searches do not accurately indicate true influenza epidemics, but epidemics of fear [11,35]. This phenomenon may also increase the database searches for oseltamivir, possibly resulting in the biased real-time detection of infectious diseases, such as seasonal influenza outbreaks. However, we do not consider the impact of this on our results to be significant.”

**Comment:** For example the difference in 1.7 in lab diagnostics between settings, but the dose checking was the same- what does this mean? What other explanations are there for this?

**Response:** Thank you for your comment. We have re-checked the absolute numbers of diagnoses in primary and specialized care, and there are typos in the specialized care diagnoses (Table 1). Diagnoses in years 2012-16 equal 2,837 (not 6,181). Specialized care diagnoses in the whole column are the sum of influenza A and B columns (this is incorrect). These have now been revised. However, the MEM analysis has been run by using the corrected data as can be seen in Figures 1-3 (number of diagnoses on y axes similar). This typo in Table 1 does not affect the MEM results and MEM conclusions, but we have now revised the Discussion section that specialized care diagnoses would have appeared too high (6,181) compared to primary care (3,717). New conclusions have been added. We thank the reviewer for rigorous evaluation of the text.

We have revised the text as follows: “We found that influenza diagnoses in primary care appeared slightly higher compared to specialized care (3,717 vs. 2,837) since the searches in primary and specialized care appeared mainly similar (5,281 vs. 5,658) (Table 1). This could mean that HCPs encounter children with influenza and search for oseltamivir in both sectors, but due to the large number of public primary care units (health care centers) in Finland, which more children attend, primary care HCPs may diagnose more children with influenza.”

**Comment:** Could anyone be looking up the dose of oseltamivir for other reasons (ie teaching/learning?)

**Response:** This is an important point. We have added the following sentence in the Discussion section: “Some searches may have been performed by medical students for learning purposes or senior physicians in teaching situations. In addition, HCPs other than physicians (e.g., nurses, pharmacists) may have searched for information on oseltamivir. However, we assume that the majority of oseltamivir searches occur in practice in primary and specialized care by clinical physicians.”

**Comment:** Could people be prescribing without looking up the dose once they know it?

**Response:** This is possible. We have added the following sentence in Discussion: “It is also worth noting that some HCPs may be familiar with the correct oseltamivir dosage once verified, thus a database search is not performed, especially when a physician regularly encounters children with similar weights or ages.”

**Comment:** The abstract doesn't read properly, and could do with checking for basic English.

**Response:** We have proofread the manuscript throughout, especially the abstract. The text has been language-checked, edited, and proofread in terms of proper English language. Revisions in English language can be seen in the file labeled “Revised Manuscript with Tracked Changes”.
